# The effect of kidney function on the urate lowering effect and safety of increasing allopurinol above doses based on creatinine clearance: a post hoc analysis of a randomized controlled trial

**DOI:** 10.1186/s13075-017-1491-x

**Published:** 2017-12-21

**Authors:** Lisa K. Stamp, Peter T. Chapman, Murray Barclay, Anne Horne, Christopher Frampton, Paul Tan, Jill Drake, Nicola Dalbeth

**Affiliations:** 1 Department of Medicine, University of Otago, Christchurch, P. O. Box 4345, Christchurch, 8140 New Zealand; 2 Department of Rheumatology, Immunology and Allergy, Christchurch Hospital, Private Bag 4710, Christchurch, 8140 New Zealand; 3 Department of Medicine, University of Auckland, Private Bag 92019, Auckland, New Zealand

**Keywords:** Allopurinol, Chronic kidney disease, Gout, Serum urate

## Abstract

**Background:**

The use of allopurinol in people with chronic kidney disease (CKD) remains one of the most controversial areas in gout management. The aim of this study was to determine the effect of baseline kidney function on safety and efficacy of allopurinol dose escalation to achieve serum urate (SU) <6 mg/dl.

**Methods:**

We undertook a post hoc analysis of a 24-month allopurinol dose escalation treat-to-target SU randomized controlled trial, in which 183 people with gout were randomized to continue current dose allopurinol for 12 months and then enter the dose escalation phase or to begin allopurinol dose escalation immediately. Allopurinol was increased monthly until SU was <6 mg/dl. The effect of baseline kidney function on urate lowering and adverse effects was investigated.

**Results:**

Irrespective of randomization, there was no difference in the percentage of those with creatinine clearance (CrCL) <30 ml/min who achieved SU <6 mg/dl at the final visit compared to those with CrCL ≥30 to <60 ml/min and those with CrCL ≥60 ml/min, with percentages of 64.3% vs. 76.4% vs. 75.0%, respectively (*p* = 0.65). The mean allopurinol dose at month 24 was significantly lower in those with CrCL <30 ml/min as compared to those with CrCL ≥30 to <60 ml/min or CrCL ≥60 ml/min (mean (SD) 250 (43), 365 (22), and 460 (19) mg/day, respectively (*p* < 0.001)). Adverse events were similar among groups.

**Conclusions:**

Allopurinol is effective at lowering urate even though and accepting that there were small numbers of participants with CrCL <30 ml/min, these data indicate that allopurinol dose escalation to target SU is safe in people with severe CKD. The dose required to achieve target urate is higher in those with better kidney function.

**Trial registration:**

Australian and New Zealand Clinical trials Registry, ACTRN12611000845932. Registered on 10 August 2011.

## Background

The use of allopurinol in people with chronic kidney disease (CKD) remains one of the most controversial areas in gout management. While recommendations from the American College of Rheumatology (ACR) [1] and European League Against Rheumatism (EULAR) [2] both advocate allopurinol as a first-line urate lowering therapy (ULT), for dosing guidance the ACR advocates a gradual dose escalation even in those with CKD [1], while EULAR recommends dose restriction based on creatinine clearance (CrCL) [2]. This discrepancy is due to concerns over increased risk of adverse effects, particularly allopurinol hypersensitivity syndrome (AHS) and limited data on the use of allopurinol in CKD.

Since the initial allopurinol dosing guidelines by Hande et al., based on an association between allopurinol dose, oxypurinol and AHS, were published [3], a number of other risk factors for AHS have been identified [4]. The Hande dosing strategy does not differentiate between starting dose, which has been associated with AHS [5], and maintenance dose (i.e. dose required to achieve target serum urate (SU)). Once AHS occurs, people with CKD have higher mortality [6].

Clinical trials of ULT have excluded those with significant CKD and restricted the allopurinol dose to ≤ 300 mg daily even in those with normal kidney function [7–9]. We have previously reported results from a randomized controlled trial of allopurinol dose escalation to achieve target urate in which 52% of the 183 participants had CrCL <60 ml/min, suggesting that such an approach is effective and safe [10]. Herein we undertook a post hoc analysis to determine the effect of baseline kidney function on the safety and efficacy of the allopurinol dose escalation strategy.

## Methods

### Study design

A 24-month open, randomized, controlled, parallel-group, comparative clinical trial was undertaken (ACTRN12611000845932). Ethical approval was obtained from the Multi-Regional Ethics Committee, New Zealand. Written informed consent was obtained from each participant. Full methods have been reported previously [10, 11]. In brief, 183 people with gout as defined by the American Rheumatism Association 1977 preliminary classification criteria for gout [12], receiving at least the CrCl based dose of allopurinol for  ≥ 1 month and with SU ≥6 mg/dl were recruited. People with a history of intolerance to allopurinol and those receiving azathioprine were excluded. Chronic kidney disease was not an exclusion criterion. Participants were randomized to continue current dose allopurinol for 12 months and then enter the dose escalation phase (control/DE) or to begin allopurinol dose escalation immediately (DE/DE). Allopurinol was increased monthly until SU was <6 mg/dl; in those with CrCL ≤60 ml/min allopurinol was increased by 50 mg increments and in those with CrCL >60 ml/min it was increased by 100 mg. Participants were not stratified by renal function at randomization. For the purposes of this post hoc analysis, participants were grouped according to kidney function at baseline as having (1) none/mild impairment, CrCL ≥60 ml/min (CKD stage 1 and 2), (2) moderate impairment, CrCL ≥30 to <60 ml/min (CKD stage 3) and (3) severe impairment, CrCL <30 ml/min (CKD stage 4 and 5).

### Adverse and serious advent event reporting

Treatment-emergent adverse events (AE) were defined as any AE occurring after entry into the study until the end of month 24. Worsening laboratory-defined AEs were those where there was an increase in AE grade from baseline between month 12 and month 24, using the Common Terminology Criteria for Adverse Events (CTCAE v4.0).

### Study outcomes

The primary efficacy outcome was reduction in SU at the final visit (month 24 or the final visit for those deceased or lost to follow up). Secondary efficacy outcomes included (1) the proportion of participants reaching target SU levels from baseline to months 12 and 24 and from months 12 to 24, (2) the percentage reduction in SU from baseline to months 12 and 24 and from months 12 to 24 and (3) the dose of allopurinol required to achieve SU <6 mg/dl. The primary safety outcome was serious adverse events (SAEs) and treatment-emergent or worsening AEs related to liver or kidney function.

### Statistical analysis

Baseline demographics and clinical features were summarized using standard descriptive statistics including mean, standard deviation (SD), range, frequency and percent as appropriate.

Changes from baseline to months 12 and 24 and from month 12 to month 24, and levels at 12 and 24 months were compared between kidney function groups using analysis of variance (ANOVA), which included the randomized group and the interaction between the randomized group and kidney function groups as factors. Comparisons of baseline levels were compared using one-way ANOVA, which only included kidney function group as the factor. Dichotomous outcome measures were compared using logistic regression, which included CrCL and randomized group and the interaction between the randomized group and kidney function groups as factors. A two-tailed *p* value <0.05 was taken to indicate statistical significance.

## Results

### Demographics

There were 183 participants who entered the study; 93 in the control/DE group (*n* = 14 with CrCL <30 ml/min; *n* = 31 with CrCL ≥30 to <60 ml/min; *n* = 48 with CrCL ≥60 ml/min 48) and 90 in the DE/DE group (*n* = 10 with CrCL <30 ml/min; *n* = 40 with CrCL ≥30 to <60 ml/min; *n* = 40 with CrCL ≥60 ml/min). There were 143 participants who completed the month-12 visit; 73 in the control/DE group (*n* = 8 with CrCL <30 ml/min; *n* = 24 with CrCL ≥30 to <60 ml/min; *n* = 41 with CrCL ≥60 ml/min) and 70 in the DE/DE group (*n* = 7 with CrCL <30 ml/min; *n* = 35 with CrCL ≥30 to <60 ml/min; *n* = 28 with CrCL ≥60 ml/min). There were 137 participants who completed the month-24 visit; 68 in the control/DE group (*n* = 7 with CrCL <30 ml/min; *n* = 22 with CrCL ≥30 to <60 ml/min; *n* = 39 with CrCL ≥60 ml/min) (73.1%) and 69 in the DE/DE group (*n* = 7 with CrCL <30 ml/min; *n* = 33 with CrCL ≥30 to <60 ml/min; *n* = 29 with CrCL ≥60 ml/min). Demographics for each of the randomized groups according to baseline kidney function group are outlined in Table 1.

**Table 1:** Participant baseline demographics and clinical features

Variable	Control/dose escalation (*n* = 93)			Dose escalation/dose escalation (*n* = 90)		
	CrCL <30 ml/min (*n* = 14)	CrCL ≥30 to <60 ml/min (*n* = 31)	CrCL ≥60 ml/min (*n* = 48)	CrCL <30 ml/min (*n* = 10)	CrCL ≥30 to <60 ml/min (*n* = 40)	CrCL ≥60 ml/min (*n* = 40)
Age years^a^	68.2 (14.2)	66.6 (9.3)	54.8 (11.9)	66.8 (12.5)	64.6 (9.6)	52.5 (12.1)
Male, *n* (%)	7 (50%)	25 (80.6%)	46 (95.8)	9 (90%)	34 (85%)	39 (97.5)
Ethnicity, *n* (%)						
NZ European	6 (42.9%)	13 (41.9%)	20 (41.7%)	2 (20%)	21 (52.5%)	14 (35%)
Maori	3 (21.4%)	10 (32.3%)	9 (18.8%)	3 (30.0%)	13 (32.5%)	13 (32.5%)
Pacific Island	4 (28.6%)	6 (19.4%)	17 (35.4%)	5 (50%)	5 (12.5%)	9 (22.5%)
Asian	1 (7.1)	2 (6.5%)	1 (2.1%)	0 (0%)	1 (2.5%)	4 (10.0%)
Other	0 (0%)	0 (0%)	1 (2.1%)	0 (0%)	0 (0%)	0 (0%)
Duration of gout (years)	16.8 (14.8)	18.2 (14.7)	18.1 (11.9)	13.1 (11.2)	16.9 (11.2)	16.9 (11.2)
Baseline serum urate mg/dl^a^	8.3 (1.5)	7.1 (1.6)	6.8 (1.5)	8.0 (1.6)	7.6 (1.6)	6.5 (1.3)
CrCL (ml/min)	19.8 (5.9)	44.3 (7.9)	82.4 (16.6	21.1 (6.7)	44.5 (8.1)	85.5 (17.7)
Body mass index (kg/m^2^)^a^	34.6 (7.2)	35.8 (8.3)	35.1 (7.5)	36.9 (8.4)	35.9 (8.4)	33.7 (6.8)
Baseline allopurinol dose mg/day^b^	135.7 (100-250)	258.1 (150-400)	328.1 (200-600)	160.0 (100-300)	231.9 (100-600)	317.5 (150-600)
Allopurinol dose, *n* (%)						
≤ 200 mg/day	13 (92.9%)	13 (41.9%)	5 (10.4%)	9 (90%)	25 (62.5%)	3 (7.5%)
> 200–300 mg/day	1 (7.1%)	16 (51.6%)	33 (68.8%)	1 (10%)	13 (32.5%)	32 (80%)
> 300 mg/day	0 (0%)	2 (6.5%)	10 (20.8%)	0 (0%)	2 (5%)	7 (7.8%)
Presence of palpable tophi, *n* (%)	10 (71.4%)	14 (45.2%)	22 (45.8%)	4 (40%)	13 (32.5%)	18 (45%)
Co-existing conditions, *n* (%)						
Obesity^c^	11 (78.6%)	23 (74.2%)	36 (75%)	8 (80%)	29 (72.5%)	27 (67.5%)
Kidney stones	0 (0%)	1 (3.2%)	2 (4.2%)	1 (10%)	3 (7.5%)	1 (2.5%)
Cardiovascular disease^d^	13 (92.9%)	14 (45.2%)	11 (22.9%)	5 (50%)	26 (65%)	10 (25%)
Diabetes mellitus	8 (57.1%)	12 (38.7%)	13 (27.1%)	7 (70%)	18 (45%)	4 (10%)
Hypertension	11 (78.6%)	29 (93.5%)	25 (52.1%)	9 (90%)	36 (90%)	22 (55%)
Hyperlipidemia	12 (85.7%)	19 (61.3%)	27 (56.3%)	7 (70%)	22 (55%)	18 (45%)
Concurrent medications, *n* (%)						
Diuretic	13 (92.9)	19 (61.3%)	11 (22.9%)	7 (70%)	23 (57.5%)	8 (20.0%)
Aspirin	11 (78.6%)	17 (54.8%)	13 (27.1%)	7 (70%)	23 (57.5%)	10 (25%)
Any anti-inflammatory prophylaxis	5 (35.7%)	15 (48.4%)	25 (52.1%)	4 (40%)	24 (60%)	23 (57.5%)
Colchicine	2 (14.3%)	11 (25.5%)	22 (45.8%)	3 (30%)	13 (32.5%)	18 (45%)
NSAID	0 (0%)	3 (9.7%)	6 (12.5%)	2 (20%)	4 (10%)	9 (22.5%)
Prednisone	3 (21.4%)	8 (19.4%)	3 (6.3%)	1 (10%)	9 (22.5%)	2 (5%)

*CrCL* creatinine clearance, *NSAID* non steroidal anti-inflammatory drug

^a^Mean (SD)

^b^Mean (range)

^c^Obesity defined as body mass index ≥30 kg/m^2^

^d^Cardiovascular disease defined as ischemic heart disease, heart failure or peripheral vascular disease

### Serum urate

**Fig. 1:**
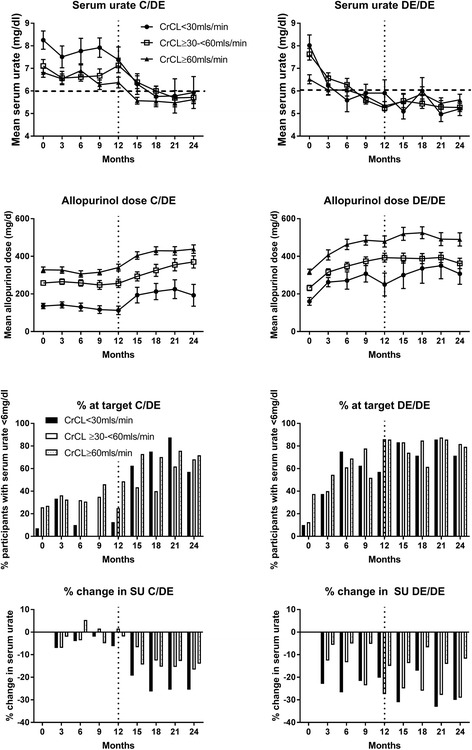
Mean serum urate, time course for achieving target serum urate (SU), mean percentage change in SU over the 24-month study period and mean allopurinol dose in the control/dose escalation phase(C/DE) and immediate dose escalation (DE/DE) groups by kidney function group. The vertical line represents the start of the open-label extension phase of the study. CrCL, creatinine clearance

In both the control/DE and DE/DE groups the mean baseline SU was significantly higher in those with CrCL <30 ml/min compared to those with CrCL ≥30 to <60 ml/min and ≥60 ml/min (Fig. 1a). Mean SU was below 6 mg/dl in all kidney function groups by month 24 (Fig. 1a). The percentage with SU <6 mg/dl at the final visit by randomization and by kidney function groups is shown in Fig. 1b. Irrespective of randomization, there was no significant difference in the percentage of those with CrCL <30 ml/min who achieved SU <6 mg/dl at the final visit compared to those with CrCL ≥30 to <60 ml/min and ≥60 ml/min, with percentages of 64.3% vs. 76.4% vs. 75.0%, respectively (*p* = 0.65). The mean (standard error (SE)) change in SU from baseline to month 24 irrespective of randomization, was significantly higher in those with CrCL <30mls/min; mean change -2.23 (0.88) mg/dl in those with CrCL <30mls/min, -1.98 (0.23) mg/dl in those with CrCL ≥30 to <60 ml/min and -1.00 (0.79) mg/dl in those with CrCL ≥60 ml/min (*p* = 0.002). The mean change in SU by kidney function and randomization group is shown in Table 2.

**Table 2:** Primary and secondary efficacy endpoints

Variable	C/DE (*n* = 93)			DE/DE (*n* = 90)			*P* values^a^
	CrCL <30 ml/min (*n* = 14)	CrCL ≥30 to <60 ml/min (*n* = 31)	CrCL ≥60 ml/min (*n* = 48)	CrCL <30 ml/min (*n* = 10)	CrCL ≥30 to<60 ml/min (*n* = 40)	CrCL ≥60 ml/min (*n* = 40)	
Change in serum urate (mg/dl), mean (SE)							
Baseline to month 12	-0.67 (0.67)	-0.05 (0.33)	-0.25 (0.26)	-1.49 (0.47)	-2.27 (0.29)	-1.06 (0.24)	0.04
Baseline to month 24	-2.23 (0.88)	-1.41 (0.36)	-1.10 (0.25)	-2.47 (0.68)	-2.35 (0.28)	-0.86 (0.27)	0.15
Month 12 to month 24	-1.42 (0.45)	-1.51 (0.41)	-0.87 (0.25)	-1.20 (0.29)	0.01 (0.17)	0.34 (0.23)	0.82
Serum urate <6 mg/dl, %							
Month 12	13%	25%	49%	57%	86%	86%	0.50
Month 24	57%	68%	72%	71%	82%	79%	0.93
Mean (SE) serum urate							
Baseline	8.25 (0.41)	7.10 (0.29)	6.82 (0.21)	8.02 (0.46)	7.63 (0.26)	6.52 (0.20)	<0.001^b^
Month 12	7.38 (0.57)	7.13 (0.32)	6.37 (0.24)	5.91 (0.59)	5.25 (0.12)	5.34 (0.19)	0.20
Month 24	5.93 (0.71)	5.72 (0.25)	5.62 (0.19)	5.21 (0.30)	5.27 (0.18)	5.61 (0.25)	0.46
Percentage change in serum urate from baseline, mean (SE)							
Baseline to month 12	-6.2 (7.2)	1.6 (4.9)	-1.9 (3.4)	-20.2 (6.0)	-27.5 (2.9)	-15.0 (3.5)	0.08
Baseline to month 24	-25.5 (8.5)	-16.6 (4.8)	-13.9 (3.2)	-30.0 (5.8)	-29.1 (2.8)	-11.8 (4.1)	0.14
Month 12 to month 24	-19.4 (5.4)	-17.9 (4.6)	-10.1 (3.5)	-2.4 (5.4)	0.87 (3.2)	7.75 (4.6)	0.99
Percentage with at least one flare in preceding month							
Baseline	50%	38.7%	56.3%	40.0%	32.5%	42.5%	0.17^b^
Month 12	37.5%	12.5%	39%	14.3%	34.3%	32.1%	0.10
Month 24	14.3%	13.6%	20.5%	0.0%	15.2%	3.4%	0.30
Allopurinol dose (mg/day) to achieve target SU at month 24, mean (range)	262.5 (150–500)	389.3 (250–650)	439.3 (300–800)	350.0 (250–600)	396.3 (200–700)	491.3 (300–900)	0.002^b^
Number of participants requiring >300 mg/day to achieve target SU at month 24	1/4 (25%)	9/14 (64.3%)	20/28 (71.4%)	1/5 (20%)	16/27 (59.3%)	19/23 (82.6%)	0.013
Percentage of individuals receiving anti-inflammatory prophylaxis							
Baseline	35.7%	48.4%	52.1%	40.0%	60.0%	57.5%	0.30^b^
Month 12	37.5%	29.2%	29.3%	71.4%	34.3%	14.3%	0.15
Month 24	14.3%	18.2%	15.4%	57.1%	9.1%	10.3%	0.16
HAQ mean (SE) change							
Baseline to month 12	-0.10 (0.19)	0.10 (0.15)	-0.14 (0.09)	0.47 (0.28)	0.06 (0.12)	-0.13 (0.11)	0.25
Baseline to month 24	-0.09 (0.21)	0.14 (0.11)	-0.32 (0.07)	-0.19 (0.30)	-0.05 (0.11)	-0.20 (0.12)	0.67
Month 12 to month 24	0.10 (0.04)	-0.09 (0.18)	-0.22 (0.08)	-0.72 (0.35)	-0.05 (0.13)	-0.05 (0.11)	0.04
Pain VAS mean (SE) change							
Baseline to month 12	-0.25 (0.90)	1.04 (0.51)	-0.02 (0.40)	0.43 (0.61)	0.17 (0.40)	-0.43 (0.54)	0.57
Baseline to month 24	-1.15 (1.3)	0.27 (0.62)	-1.10 (0.35)	-0.50 (1.36)	-0.64 (0.37)	-1.28 (0.53)	0.56
Month 12 to month 24	-0.71 (0.71)	-0.52 (0.84)	-1.03 (0.37)	-0.60 (0.87)	-0.67 (0.34)	-0.78 (0.57)	0.93
SJC mean (SE) change							
Baseline to month 12	-2.88 (1.84)	-0.04 (1.05)	-0.07 (0.72)	0.86 (1.6)	-1.17 (0.89)	0.17 (0.53)	0.19
Baseline to month 24	-2.14 (1.39)	-2.86 (1.65)	-1.26 (0.73)	-0.67 (0.67)	-1.85 (0.93)	-0.24 (0.27)	0.99
Month 12 to month 24	1.23 (1.16)	-2.91 (2.24)	-1.34 (0.98)	0 (0)	-0.52 (0.42)	-0.37 (0.59)	0.57
TJC mean (SE) change							
Baseline to month 12	2.13 (3.56)	0.04 (1.33)	-2.83 (1.07)	-0.57 (0.62)	-1.57 (0.90)	1.18 (1.42)	0.04
Baseline to month 24	-2.57 (1.97)	-1.73 (1.49)	-2.08 (0.88)	-0.50 (0.34)	-0.42 (1.54)	-0.97 (0.50)	0.97
Month 12 to month 24	-5.0 (3.0)	-2.57 (1.83)	0.65 (0.55)	-0.20 (0.20)	1.18 (1.32)	-1.96 (1.37)	0.02

*C/DE* control/dose escalation phasegroup, *DD* immediate dose escalation group, *CrCL* creatinine clearance, *SU* serum urate, *HAQ* health assessment questionnaire, *VAS* visual analogue scale, *SJC* swollen joint count, *TJC* tender joint count

^a^For levels and changes after baseline the *p* value tests the significance of randomization depending on baseline CrCL

^b^For baseline assessments the *p* value represents the significance of differences between kidney function groups

The mean percentage change in SU by randomization and by kidney function groups is shown in Fig. 1c. Irrespective of randomization, the mean (SE) percentage change in SU from baseline to final visit in those with CrCL <30 ml/min was similar to those with CrCL ≥30 to <60 ml/min and significantly higher than those with CrCL ≥60 ml/min (-27.7% (5.0%) vs. -24.1% (2.7%) vs. -13.0% (2.5%) (*p* = 0.003)).

### Allopurinol dose

**Fig. 2:**
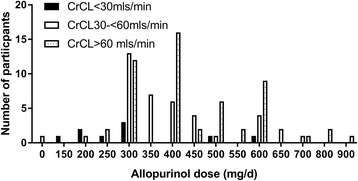
Allopurinol dose at month 24 in those with serum urate <6 mg/dl. CrCL, creatinine clearance

Mean allopurinol dose during the study period by randomization and by kidney function groups is shown in Fig. 1d. Irrespective of randomization, mean (SE) allopurinol dose at baseline was lower in those with lower CrCL; 146 (18) mg/day, 243 (10) mg/day and 323 (9) mg/day (*p* < 0.001) in those with CrCL <30mls/min, ≥30 to <60 ml/min and ≥60 ml/min, respectively. Irrespective of randomization, the mean (SE) allopurinol dose at month 24 was significantly lower in those with CrCL <30 ml/min as compared to those with CrCL ≥30 to <60 ml/min or CrCL ≥60 ml/min (250 (43) mg/day, 365 (22) mg/day and 460 (19) mg/day, respectively (*p* < 0.001)). Allopurinol dose in those with SU <6 mg/dl at month 24 is shown in Fig. 2.

The allopurinol dose required to achieve target SU was associated with baseline kidney function (Table 2). Of those with CrCL <30 ml/min (n = 14), only one participant in the control/DE and one in the DE/DE group required >300 mg/day to achieve target SU. In those with higher CrCL, the number of participants requiring >300 mg/day allopurinol to achieve target SU was higher (Table 2). Irrespective of randomization, the mean (range) allopurinol dose required to achieve target SU was higher in those with better kidney function (311.1 mg/day (150–600) vs. 393.9 mg/day (200–700) vs. 462.8 mg/day (300–900)).

### Adverse events

**Fig. 3:**
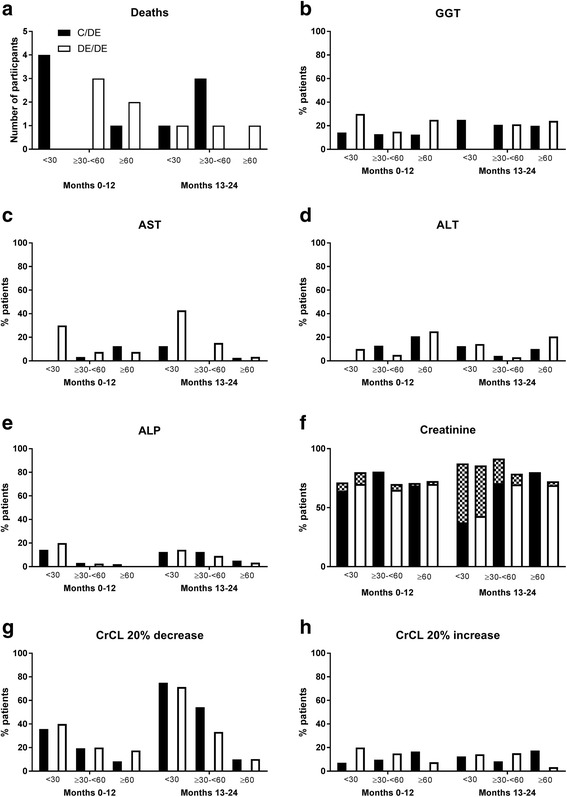
Deaths and treatment-emergent or worsening laboratory adverse events (AEs): number of individuals with at least one AE over the 24-month study period by kidney function groups in the dose escalation phase (control/DE) and immediate dose escalation (DE/DE) groups. **a** Deaths. **b**-**e** Liver function. **f** Percentage of participants with increase in creatinine from baseline (solid bars, C/DE; open bars, DE/DE, Common Terminology Criteria for Adverse Events (CTCAE) grade 1. Hatched area indicates CTCAE grade 2). **g**, **h** Percentage of participants with > 20% decrease (worsening) (**g**) or increase (improvement) (**h**) in creatinine clearance (CrCL) from baseline. GGT, gamma glutamyl transferase; AST aspartate aminotransferase; ALT, alanine transaminase; ALP, alkaline phosphatase

There were 17 deaths during the study period, details of these have been published previously [10, 11]. During the RCT phase of the study there were five deaths in the control group of which four occurred in those with CrCL <30mls/min. In comparison none of the five deaths in the dose escalation groups had a CrCL <30 ml/min (Fig. 3). During the open extension phase of the study there were four deaths in the control/DE group, of which one occurred in those with CrCL <30mls/min. In comparison, there were three deaths in the DE/DE group, of which one occurred in those with CrCL <30mls/min. Of note there were high rates of co-morbidities and in particular cardiovascular disease at baseline in those with CrCL <30 ml/min (Table 1). The number of SAEs according to kidney function group and randomization group are shown in Table 3. The type and number of SAEs was as expected and was similar between groups. The percentage of participants with treatment emergent or worsening gamma glutamyl transferase (GGT), alanine transaminase (ALT), aspartate aminotransferase (AST), alkaline phosphatase (ALP), creatinine and CrCL by kidney function groups are shown in Fig. 3.

**Table 3:** Serious adverse events during months 0–12 and months13–24 by kidney function group

CTCAE category	Time period	Control (*n* = 93)			Dose escalation (*n* = 90)		
		CrCL <30 ml/min (*n* = 14)	CrCL ≥30 to <60 ml/min (*n* = 31)	CrCL ≥60 ml/min (*n* = 48)	CrCL <30 ml/min (*n* = 10)	CrCL ≥30 to <60 ml/min (*n* = 40)	CrCL ≥60 ml/min (*n* = 40)
Cardiac disorders	Month 0–12	6 (3)	7 (4)	1 (1)	1 (1)	9 (7)	4 (3)
	Month 13–24	3 (2)	8 (5)	0	0	6 (5)	2 (2)
Gastrointestinal disorders	Month 0–12	1 (1)	3 (3)	2 (2)	0	3 (3)	0
	Month 13–24	0	5 (4)	1 (1)	0	3 (3)	0
General disorders	Month 0–12	1 (1)	0	0	0	0	1 (1)
	Month 13–24	1 (1)	0	1 (1)	0	0	1 (1)
Hepatobiliary disorders	Month 0–12	0	0	0	0	1 (1)	0
	Month 13–24	0	0	0	0	0	0
Infections and infestations	Month 0–12	2 (2)	3 (3)	3 (3)	1 (1)	2 (1)	1 (1)
	Month 13–24	0	6 (4)	2 (2)	3 (3)	5 (3)	1 (1)
Injury, poisoning and procedural complications	Month 0–12	1 (1)	0	1 (1)	0	1 (1)	0
	Month 13–24	1 (1)	3 (2)	1 (1)	0	1 (1)	0
Investigations	Month 0–12	0	0	0	0	1 (1)	0
	Month 13–24	0	0	0	0	0	0
Metabolism and nutrition	Month 0–12	0	0	0	1 (1)	1 (1)	0
	Month 13–24	0	0	0	0	0	0
Musculoskeletal	Month 0–12	0	0	1 (1)	1 (1)	0	0
	Month 13–24	0	0	2 (1)	0	1 (1)	0
Nervous system disorders	Month 0–12	1 (1)	1 (1)	1 (1)	0	0	1 (1)
	Month 13–24	3 (2)	0	0	1 (1)	3 (3)	0
Renal and urinary disorders	Month 0–12	3 (3)	2 (2)	0	1 (1)	1 (1)	0
	Month 13–24	0	0	0	2 (2)	1 (1)	0
Respiratory, thoracic and mediastinal disorders	Month 0–12	0	1 (1)	1 (1)	1 (1)	1 (1)	0
	Month 13–24	0	0	0	0	0	0
Skin and subcutaneous tissue disorders	Month 0–12	1 (1)	0	0	0	0	2 (1)
	Month 13–24	0	0	0	0	0	0
Psychiatric disorders	Month 0–12	0	0	0	0	0	0
	Month 13–24	1 (1)	0	0	0	0	1 (1)
Vascular disorders	Month 0–12	0	0	0	0	0	0
	Month 13–24	0	0	0	0	1 (1)	0

Data reported are number of events (number of individuals)

## Discussion

Long-term urate lowering in the setting of CKD is challenging and controversial. Although allopurinol is considered first-line ULT, there are limited data in those with CKD. Herein, we showed that use of allopurinol is safe and effective even in those with CKD.

There are few treatment options for urate lowering in people with stage 4 and 5 CKD and gout. There are limited data on the use of febuxostat in those with CrCL <30 ml/min [13]; uricosuric agents are either contraindicated or not effective in those with CrCL <30mls/min, and while pegloticase needs no dose adjustment in CKD, it is not widely available [14]. Thus it is important that clinicians can safely and effectively use allopurinol, given its widespread availability and low cost.

In the current study, similar proportions of participants in each kidney function group achieved target urate, suggesting the strategy is effective even in those with more severe CKD. The dose required to achieve target SU in those with CrCL <30 ml/min was ≤ 300 mg/day in the majority of participants. Doses >300 mg/day are only infrequently used in clinical practice even in those with normal kidney function, frequently due to physician inertia or concern about dose escalation [2, 15]. The data presented herein suggest that in people with CrCL <30 ml/min, allopurinol doses >300 mg/day are rarely needed to achieve target SU.

The numbers and types of SAEs were similar in both groups but as expected the number of cardiac events was higher in those with CrCL <30 ml/min even during the first 12 months when dose escalation was not undertaken [16]. While a number of abnormalities in kidney function were noted, they were similar between randomized groups over the study period.

There are several limitations to this study. There were only small numbers of participants with CrCL <30 ml/min and the study was not powered to detect the rare AHS. Further adequately powered studies of people with stage 4/5 CKD may be required to clarify the impact on clinical outcomes such as flares, activity limitation and health-related quality of life, and to confirm the safety of this approach. However, studies of sufficient size to detect the rare AHS are unlikely to be undertaken given the large numbers of participants that would be required.

## Conclusion

Allopurinol is effective at lowering urate even in those with severe CKD. The dose required to achieve target SU is higher in those with better kidney function. Accepting that there were small numbers of participants with CrCL <30 ml/min, these data indicate that allopurinol dose escalation to target SU is safe in people with CKD.

## Abbreviations

ACR: American College of Rheumatology
AE: Adverse event
AHS: Allopurinol hypersensitivity syndrome
ALP: Alkaline phosphatase
ALT: Alanine transaminase
ANOVA: Analysis of variance
AST: Aspartate aminotransferase
CKD: Chronic kidney disease
control/DE: Dose escalation phase group
CrCL: Creatinine clearance
CTCAE: Common Terminology Criteria for Adverse Events
DE/DE: Immediate dose escalation group
EULAR: European League Against Rheumatism
GGT: Gamma glutamyl transferase
HAQ: Health assessment questionnaire
N: Number
NSAID: Non steroidal anti-inflammatory drug
SAE: Serious adverse event
SD: Standard deviation
SE: Standard error
SJC: Swollen joint count
SU: Serum urate
TJC: Tender joint count
ULT: Urate lowering therapy
VAS: Visual analogue scale
